# Development and Validation of a Novel Hypoxia-Related Long Noncoding RNA Model With Regard to Prognosis and Immune Features in Breast Cancer

**DOI:** 10.3389/fcell.2021.796729

**Published:** 2021-12-16

**Authors:** Peng Gu, Lei Zhang, Ruitao Wang, Wentao Ding, Wei Wang, Yuan Liu, Wenhao Wang, Zuyin Li, Bin Yan, Xing Sun

**Affiliations:** ^1^ Department of General Surgery, Shanghai General Hospital, School of Medicine, Shanghai Jiao Tong University, Shanghai, China; ^2^ Department of Vascular Surgery, Intervention Center, Shanghai General Hospital, School of Medicine, Shanghai Jiao Tong University, Shanghai, China; ^3^ Department of Urology, Shanghai General Hospital, School of Medicine, Shanghai Jiao Tong University, Shanghai, China; ^4^ Department of Hepatobiliary Surgery, Peking University Organ Transplantation Institute, Peking University People’s Hospital, Beijing, China

**Keywords:** breast cancer, hypoxia, long noncoding RNA, prognosis, immune infiltration, nomogram

## Abstract

**Background:** Female breast cancer is currently the most frequently diagnosed cancer in the world. This study aimed to develop and validate a novel hypoxia-related long noncoding RNA (HRL) prognostic model for predicting the overall survival (OS) of patients with breast cancer.

**Methods:** The gene expression profiles were downloaded from The Cancer Genome Atlas (TCGA) database. A total of 200 hypoxia-related mRNAs were obtained from the Molecular Signatures Database. The co-expression analysis between differentially expressed hypoxia-related mRNAs and lncRNAs based on Spearman’s rank correlation was performed to screen out 166 HRLs. Based on univariate Cox regression and least absolute shrinkage and selection operator Cox regression analysis in the training set, we filtered out 12 optimal prognostic hypoxia-related lncRNAs (PHRLs) to develop a prognostic model. Kaplan–Meier survival analysis, receiver operating characteristic curves, area under the curve, and univariate and multivariate Cox regression analyses were used to test the predictive ability of the risk model in the training, testing, and total sets.

**Results:** A 12-HRL prognostic model was developed to predict the survival outcome of patients with breast cancer. Patients in the high-risk group had significantly shorter median OS, DFS (disease-free survival), and predicted lower chemosensitivity (paclitaxel, docetaxel) compared with those in the low-risk group. Also, the risk score based on the expression of the 12 HRLs acted as an independent prognostic factor. The immune cell infiltration analysis revealed that the immune scores of patients in the high-risk group were lower than those of the patients in the low-risk group. RT-qPCR assays were conducted to verify the expression of the 12 PHRLs in breast cancer tissues and cell lines.

**Conclusion:** Our study uncovered dozens of potential prognostic biomarkers and therapeutic targets related to the hypoxia signaling pathway in breast cancer.

## Background

Female breast cancer surpassed lung cancer as the most frequently diagnosed cancer (representing 11.7% of total cases and 24.5% of female cancers) worldwide in 2020 (Sung et al., 2021). Despite recent advances in high-quality prevention, early detection, and treatment services, breast cancer is still the leading cause of cancer-related death for female patients (15.5%) (Loibl et al., 2021a). Thus, it is of great importance to identify novel prognosticators and develop a more precise prognostic model to help optimize the individual treatment for patients.

Tumor cells grow in and continuously interact with an incredibly complex and dynamic network called tumor microenvironment (TME). TME consists of noncellular components, such as extracellular matrix, growth factors, cytokines, enzymes, and hormones, as well as diverse types of cells, including endothelial cells, pericytes, immune inflammatory cells, and fibroblasts (Hanahan and Weinberg, 2011). Hypoxia, as one of the most important abnormal microenvironments that tumor cells are continuously exposed to, has a significant impact on tumor biology, leading to a higher phenotypic heterogeneity (Marusyk et al., 2012). The hypoxic tissue areas of many breast cancers are heterogeneously distributed within the tumor mass (Vaupel et al., 2004). Oxygen tension (pO_2_) measured in normal breast tissue exhibited a mean (and median) of 65 mm Hg (Vaupel et al., 2007), whereas for breast cancers in T1b–T4 stages, the median pO_2_ was 28 mm Hg (Vaupel et al., 1991). The hypoxic response is mainly ascribed to HIF. The HIF is a heterodimer comprising an inducible α subunit (HIF-α) and a constitutively expressed β subunit (HIF-β), which functions as a transcriptional factor that regulates the expression of genes involved in diverse biological characteristics of tumors (Wang et al., 1995). The levels of HIF-1α have been implicated as an independent prognostic factor for patients with breast cancer (Bos et al., 2003). The hallmarks of cancer, such as angiogenesis (Niu et al., 2020), invasion and metastasis (Bristow and Hill, 2008), evading immune destruction (Hsing et al., 2012) and reprogramming of energy metabolism (Samanta and Semenza, 2018), have been validated to be tumor supportive and associated with the hypoxic tumor environment of breast cancer.

LncRNAs are defined as RNA transcripts longer than 200 nucleotides in length, with no potential to encode proteins (Kopp and Mendell, 2018). Accumulating evidence suggests that lncRNAs play crucial roles in the occurrence and development of a large variety of cancers including breast cancer (Gao et al., 2020; Liu et al., 2021). During tumor hypoxia, hypoxia-inducible factors (HIFs) are upregulated and either positively or negatively regulate lncRNAs through hypoxia response elements within their promoters. Or vice versa, some lncRNAs can regulate the HIF signaling pathway directly or indirectly (Choudhry et al., 2016). Moreover, increasing evidence reveals the potential of lncRNAs as diagnostic or prognostic biomarkers (Bin et al., 2018; Qian et al., 2020; Chen Y. et al., 2021).

Though there was a similar study proposing a prognostic signature based on HRLs (Zhao et al., 2021), which served to stratify patients with early-stage breast cancer, we aimed to develop and validate a more comprehensive and reliable prognostic model for predicting the survival of all breast cancer patients. By means of both bioinformatic analysis and experimental validation, we were also meant to mine some potential tumor-supportive or tumor-suppressive lncRNAs to indicate the further research direction on their functions in breast cancer.

## Materials and Methods

### Data Acquisition

We downloaded RNA sequencing data (1,109 breast cancer tissues and 113 matched normal tissues) and corresponding clinical and pathological information of patients with breast cancer from The Cancer Genome Atlas (TCGA) (https://gdc.cancer.gov/) database in March 2021. The clinical characteristics of the patients are listed in Table 1. A total of 200 hypoxia-related mRNAs were obtained from the Molecular Signatures Database V7.3 (Subramanian et al., 2005) (https://www.gsea-msigdb.org/gsea/msigdb/, M5891).

**TABLE 1:** Baseline patients characteristics (n = 1,097).

Characteristic	N (1,097)	%
Age		
≤65 years	776	70.7
>65 years	321	29.3
Unknown	0	0
Sex		
Female	1,085	98.9
Male	12	1.1
Unknown	0	0
Clinical Stage		
I	183	16.7
II	621	56.6
III	249	22.7
IV	20	1.8
Unknown	24	2.2
Pathologic T Stage		
T1	281	25.6
T2	635	57.9
T3	138	12.6
T4	40	3.6
Unknown	3	0.3
Pathologic N Stage		
N0	516	47.0
N1	364	33.2
N2	120	10.9
N3	77	7.0
Unknown	20	1.8
Pathologic M Stage		
M0	912	83.1
M1	22	2.0
Unknown	163	14.9
ER status		
Positive	808	73.7
Negative	238	21.7
Unknown	51	4.6
PR status		
Positive	699	63.7
Negative	344	31.4
Unknown	54	4.9
HER2 status		
Positive	149	13.6
Negative	640	58.3
Unknown	308	28.1
Molecular subtypes		
Luminal A/B	501	45.7
HER2 positive	149	13
TNBC	139	12.7
Unknown	308	28.1

### Screening of Hypoxia-Related lncRNAs

Differentially expressed hypoxia-related mRNAs (DEHmRNAs) between the breast cancer and normal tissues were identified via the differential expression analysis using the R package “DEseq” with the threshold value set as |log_2_ fold change (FC)| >1 and adjusted *p* value of <0.05. Heatmap and volcano plots were drawn using the R packages “pheatmap” and “EnhancedVolcano,” respectively. The PPI (protein–protein interaction) network was constructed using the STRING version 11.0 Program (Szklarczyk et al., 2017). Further, we performed Gene Ontology (GO) functional enrichment analysis and Kyoto Encyclopedia of Genes and Genomes (KEGG) pathway enrichment analysis via the R package “clusterProfiler”. Finally, we identified the HRLs by Spearman correlation analysis of DEHmRNAs according to the criteria of |Correlation Coefficient| > 0.4 and *p* < 0.001. The mRNAs–lncRNAs network were constructed via Cytoscape 3.8.2.

### Construction of a Hypoxia-Related lncRNA Prognostic Model

Univariate Cox regression analysis was used to identify prognostic hypoxia-related lncRNAs via the R package “survival” with *p* < 0.05 as the criteria. Heatmap and box plot were drawn using the R packages “pheatmap” and “ggplot2,” respectively. Spearman’s rank correlation was performed to assess the correlation among these genes using the R package “corrplot.” Then, we randomly divided patients into a training set and a testing set in the ratio of 1:1 using the R package “caret.” The R package “glmnet” was used to perform least absolute shrinkage and selection operator (LASSO) Cox regression analysis in the training set to generate a coefficient for each hypoxia survival-related lncRNA. Then, the risk score for each sample was calculated based on the formula: *Risk Score (RS) =* $\overset{n}{\underset{i=1}{\sum }}\left(Expi*Coefi\right)$ *.* Coef means the coefficient of lncRNAs associated with survival, and Exp means the expression of lncRNAs. Hence, breast cancer samples were divided into a high-risk group and a low-risk group according to the median risk score.

### Validation of the Model

Kaplan–Meier survival analysis was conducted to investigate the differences in OS and DFS between high- and low-risk groups using the R packages “survival” and “survminer,” no matter whether we classified the patients into different groups based on their clinicopathological characteristics. Receiver operating characteristic (ROC) curves and area under the curve (AUC) were used to evaluate the sensitivity and specificity of this prognostic model via the R package “survivalROC.” Additionally, we used “survival” R package to perform univariate and multivariate Cox regression analyses so as to estimate the prognostic value of the model constructed. Finally, we formulated a nomogram using the R package “rms,” which could assign points for independent prognostic factors to predict 1-, 3-, 5-, and 10-year OS of individual patients with breast cancer (Sui et al., 2020). Meanwhile, we calculated the concordance index (C-index) using “survcomp” and constructed calibration curves to evaluate the predictive power of the nomogram.

### Drug Sensitivity Analysis

We used the R package “pRRophetic” to predict the differences in chemosensitivity from tumor gene expression levels for some chemotherapy drugs, which was decided by the half-maximal inhibitory concentration (IC50), between high- and low-risk groups based on our prognostic model (Geeleher et al., 2014).

### Gene Set Enrichment Analysis

We performed gene set enrichment analysis (GSEA) (version 4.1.0, https://www.gsea-msigdb.org/gsea/index.jsp) to identify the related differential biological function and pathways between high- and low-risk groups as separated by the prognostic model using the following gene sets: Hallmark, GO, KEGG and BioCarta. Each analysis included 1,000 random permutations. The statistical significance level was set to be *p* < 0.05 and the false discovery rate as < 0.25. The plots were drawn using the R package “ggplot2.”

### Immune Cell Infiltration Analysis

The CIBERSORT(Floberg et al., 2021), CIBERSORT-ABS(Yuan et al., 2021), TIMER (tumor immune estimation resource) (Jiang et al., 2021), xCELL (Sui et al., 2020), quanTIseq (Subramanian et al., 2005), EPIC (extended polydimensional immunome characterization) (Yeo et al., 2020), MCPcounter (Dienstmann et al., 2019) algorithms were used to further analyze the differences in immune cell infiltration between high- and low-risk groups. The immune score for each single sample was calculated using the R package “estimate.” Additionally, we calculated the correlation between infiltrating immune cells and risk scores using Spearman’s rank correlation with the criteria of *p* < 0.05. Subsequently, 11 immune checkpoints were selected from the previous literature (Marin-Acevedo et al., 2018).

### RNA Extraction and Real-Time Quantitative PCR

Total RNA was extracted from 11 breast cancer tissues, paired adjacent normal tissues, and breast cell lines including MCF10A and MDA-MB-231 (under normoxia or hypoxia conditions) cell lines. RT-qPCR experiments were performed as previously described. The primers used in this study were shown in Supplementary Table S1. The mRNA quantification of the 12 PHRLs was based on the 2^–∆∆Ct^ method and the expression levels were plotted by using *ACTB* as the reference gene.

### Statistical Analysis

Data were processed using Perl language (version 5.26.1) and analyzed using R software (version 1.3.1073). The Wilcoxon test was applied to compare gene expression, risk scores, drug sensitivity (IC50), and immune cell infiltration scores between two independent groups. Univariate Cox regression and LASSO Cox regression analyses were applied to identify the most useful lncRNAs to build a prognostic model. Survival analysis was conducted using the Kaplan-Meier method and log-rank tests. The chi-square test was used to compare the relationship between risk and immune score level and other categorical clinicopathological factors. Correlation analysis was assessed by Spearman’s rank correlation. Statistical significance was set at *p* < 0.05 or 0.001 for diverse analysis.

## Results

### Identification of Differentially Expressed Hypoxia-Related mRNAs in Breast Cancer

**FIGURE 1:**
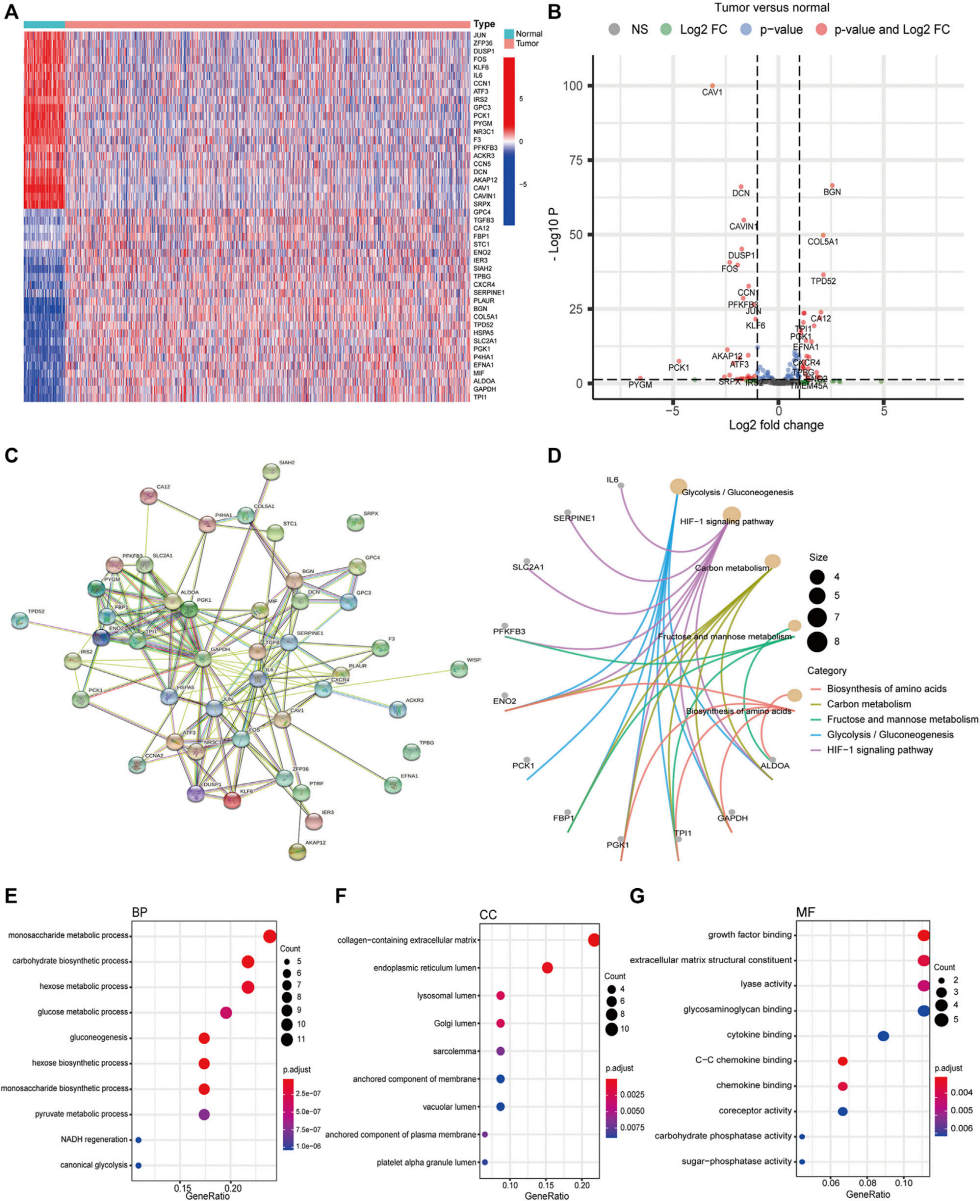
Differential expression and functional annotation of DEHmRNAs in breast cancer. **(A)** Heatmap and **(B)** volcano plot of DEHmRNAs between breast cancer tissues and normal tissues (HTSeq-Counts data from TCGA, “DESeq”). **(C)** A PPI network of DEHmRNAs. **(D)** KEGG pathway and **(E–G)** GO function enrichment analysis of DEHmRNAs.

We obtained 200 hypoxia-related mRNAs from the Molecular Signatures Database V7.3 (HALLMARK_HYPOXIA, M5891), which were previously validated to be hypoxia-regulated in multiple kinds of cancer including breast cancer (Wang et al., 2009; Xia and Kung, 2009; Leithner et al., 2014). By comparing their expression levels between 1,109 breast cancer tissues and 113 normal tissues using the R package “DEseq,” we identified 46 DEHmRNAs (Supplementary Table S2). The heatmap and volcano plot are shown in Figures 1A,B. A PPI network showed that almost all proteins encoded by the aforementioned genes interacted with each other except SRPX and TPBG (Figure 1C). Further, we performed KEGG (Figure 1D) and GO (Figures 1E–G) analyses to investigate the biological function of 46 DEHmRNAs. The top 3 most enriched KEGG pathways were “HIF-1 signaling pathway”, “Glycolysis/Gluconeogenesis”, and “Carbon metabolism”.

### Screening of Hypoxia-Related lncRNAs and Construction of a Co-Expression Network

Co-expression analysis between DEHmRNAs and lncRNAs based on Spearman’s rank correlation was performed to screen out 166 HRLs according to the criteria of |Correlation Coefficient| >0.4 and *p* < 0.001 (Supplementary Table S3), of which 46 were significantly upregulated and 41 were downregulated (Supplementary Figure S1 and Supplementary Table S4). Based on the mRNA–lncRNA co-expression pattern, we constructed a network to show the relationships among them (Supplementary Figure S2).

### Development of Hypoxia-Related lncRNA Prognostic Model

**FIGURE 2:**
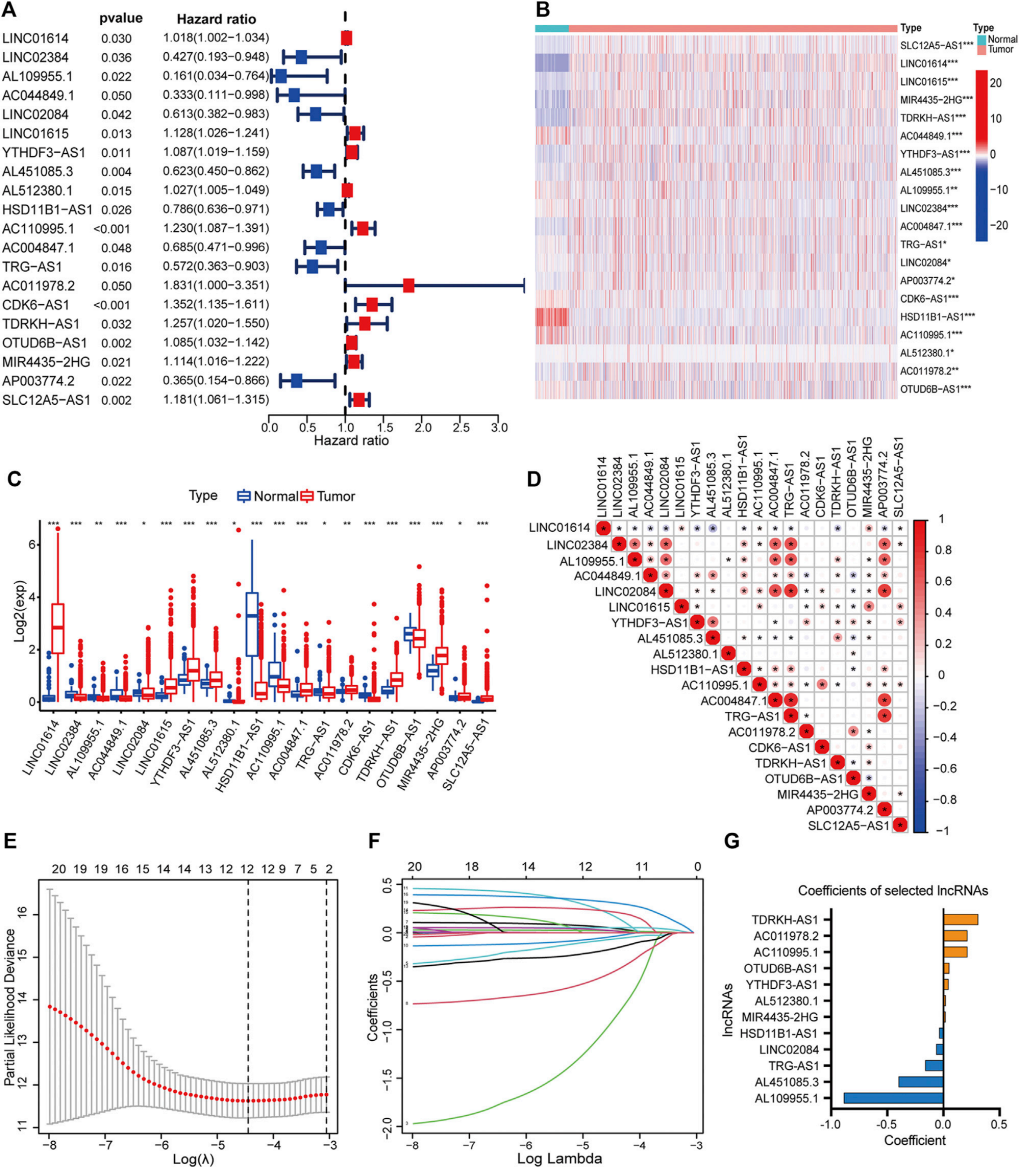
Development of a hypoxia-related lncRNA prognostic model. **(A)** Forest plot showed the hazard ratio (HR, 95% CI) and the *p* value of 20 PHRLs by univariate Cox regression analysis. Heatmap **(B)** and box plot **(C)** showed the expression level of the 20 PHRLs in breast cancer tissues compared with normal tissues (Wilcoxon test). **(D)** Spearman rank’s correlation analysis based on the expression of the 20 PHRLs. **(E and F)** LASSO-penalized Cox regression was performed to filter out 12 optimal PHRLs. **(G)** 12 PHRLs and their coefficients. *p < 0.05, **p < 0.01, and ***p < 0.001.

By matching the sample IDs in the expression matrix and clinical information profile, we collected 1,090 samples for subsequent analysis after excluding 19 samples. We performed univariate Cox regression analysis to identify 20 PHRLs significantly associated with survival (*p* < 0.05) (Figure 2A). The differential expression of these 20 PHRLs is shown in Figures 2B,C. Most of them were notably correlated with each other (Figure 2D). Then, we randomly divided these 1,090 patients with breast cancer into two groups in the ratio of 1:1: a training set (*n* = 546) and a testing set (*n* = 544). LASSO-penalized Cox regression was performed in the training set, and finally a prognostic model was developed, which consisted of seven risk lncRNAs and five protective lncRNAs on the basis of the coefficient value (Figures 2E–G). We calculated the risk scores for all samples using the following formula: Risk score = (0.309031851 × Exp of TDRKH-AS1) + (0.213481355 × Exp of AC011978.2) + (0.170772984 × Exp of AC110995.1) + (0.051734285 × Exp of OTUD6B-AS1) + (0.046646469 ×Exp of YTHDF3-AS1) + (0.019875191 × Exp of AL512380.1) + (0.019087737 × Exp of MIR4435-2HG) + (–0.037067764 × Exp of HSD11B1-AS1) + (–0.063799579 × Exp of LINC02084) + (–0.162152639 × Exp of TRG-AS1) + (–0.399031951 × Exp of AL451085.3) + (–0.886350422 × Exp of AL109955.1). All the patients were further separated into high- and low-risk groups according to the median risk score in the training set.

### Validation of the Prognostic Model

**FIGURE 3:**
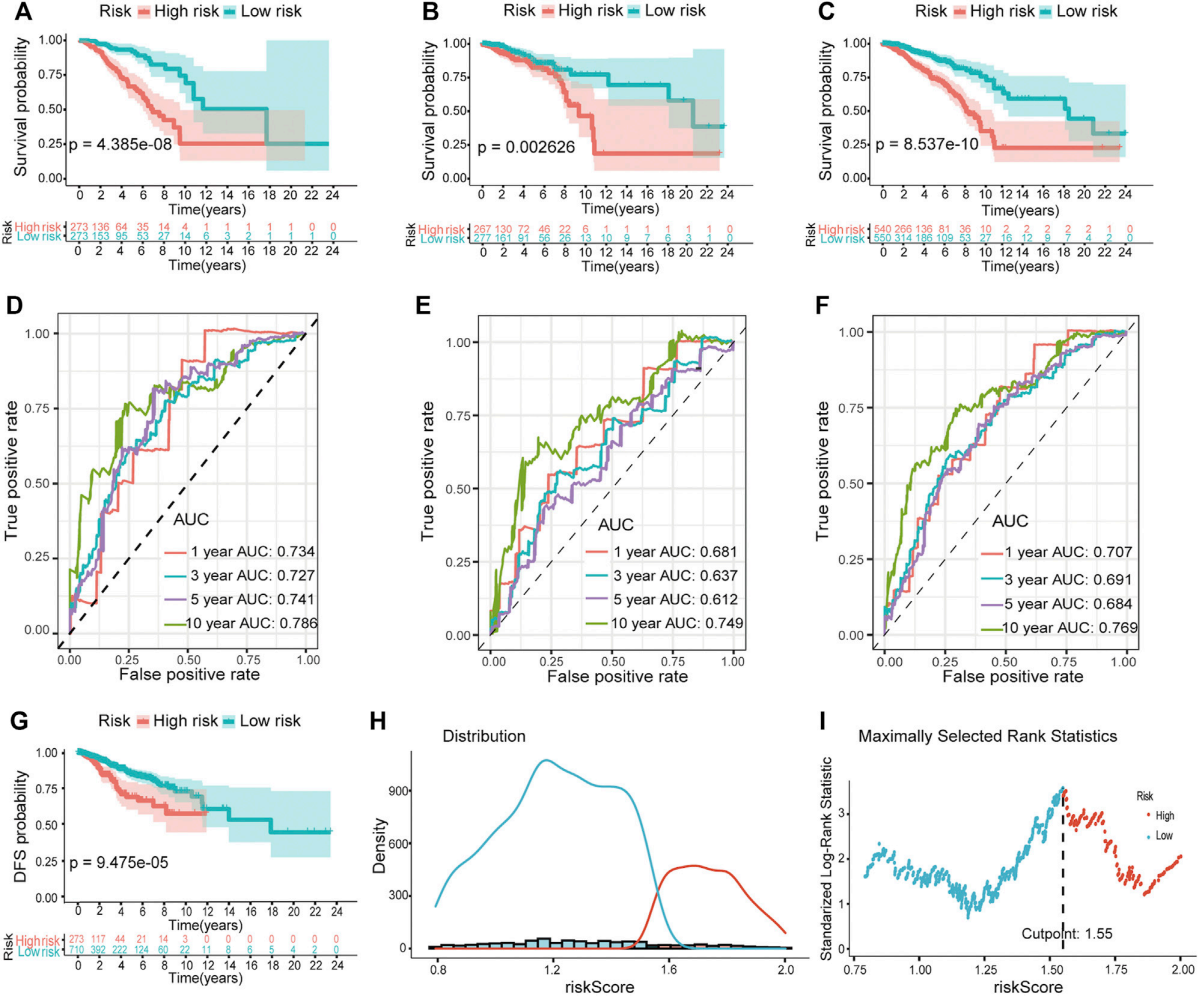
Validation of the predictive ability of the hypoxia-related lncRNA prognostic model. Kaplan–Meier survival analysis revealed the differences in survival outcomes between high- and low-risk groups in the training **(A)**, testing **(B)**, and total sets **(C)**. Time-dependent ROC analysis for evaluating the sensitivity and specificity of this prognostic model in the training **(D)**, testing **(E)**, and total sets **(F)**. **(G)** Survival curves of DFS between high- and low-risk groups. **(H and I)** The best cutpoint of the risk score for **(G)**.

We performed Kaplan–Meier survival analysis to investigate whether there any differences in survival outcomes existed between the high and low-risk groups. The results shown in Figure 3A indicated that in the training set, patients in the high-risk group had shorter median OS compared with those in the low-risk group (log-rank test, *p* = 4.385e-08). This significant difference can also be seen in both the testing set and the total set (*p* = 0.0026 in the former, *p* = 8.537e-10 in the latter) (Figures 3B,C). Then we built time-dependent ROC curves for the three sets. The AUC for 1-, 3-, 5-, and 10-year OS was 0.734, 0.727, 0.741, and 0.786 in the training set and 0.681, 0.637, 0.612, and 0.749 in the testing set, respectively (Figure 3D). In the total set, it was 0.707, 0.691, 0.684, and 0.769 (Figures 3E,F). Additionally, we compared the DFS of 984 patients with breast cancer. As expected, the patients in the high-risk group had significantly shorter median DFS than those in the low-risk group when the best cut point of the risk score was set as 1.55 (Figures 3G–I).

**FIGURE 4:**
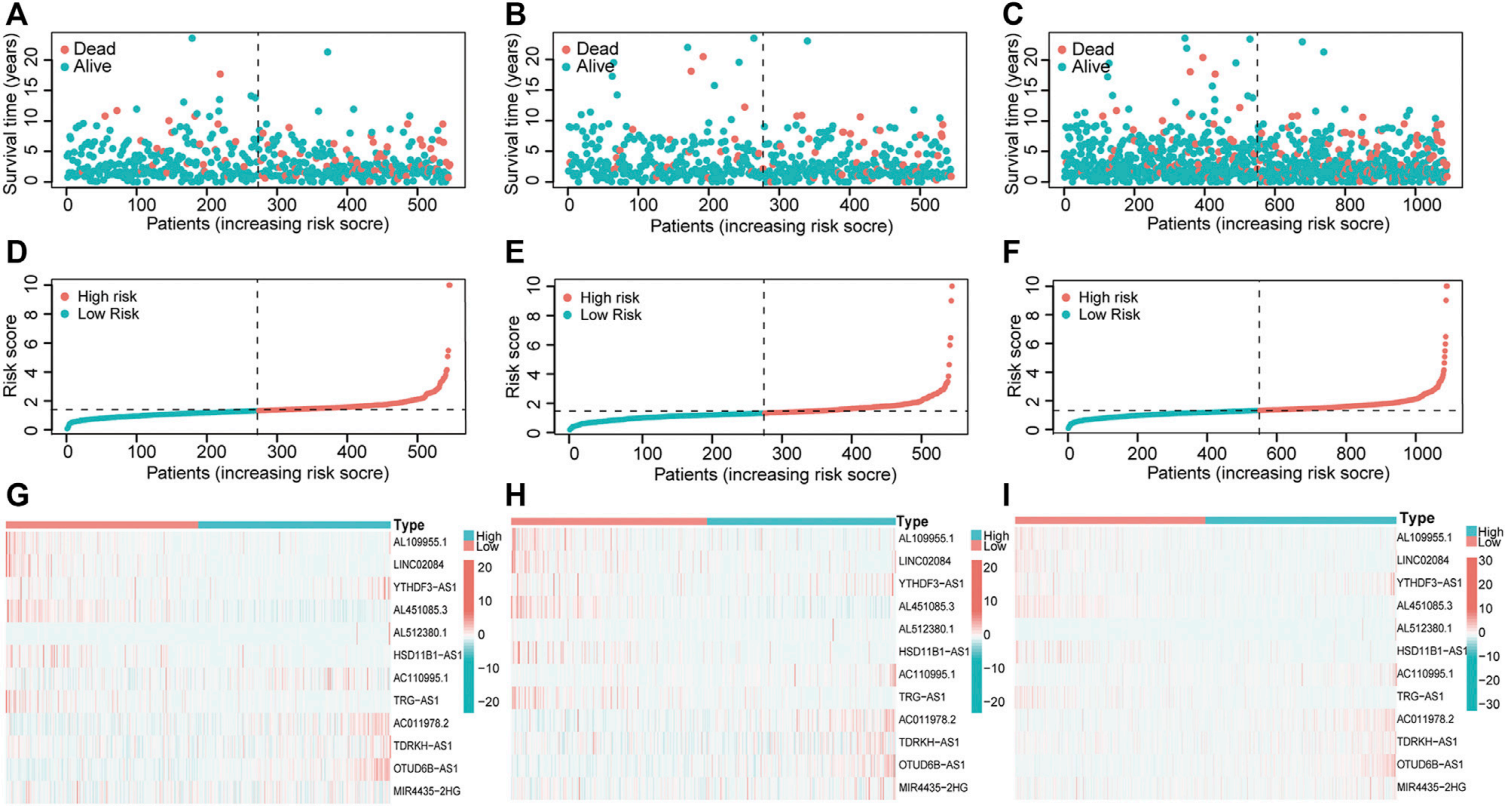
Risk score distribution, survival status, and 12 PHRLs expression. **(A,D,G)** Training set. **(B,E,H)** Testing set. **(C,F,I)** Total set.

We next visualized risk score distribution, survival status, and PHRLs expression in all three sets. The plots showed that, whether in the training (Figures 4A,D), testing (Figures 4B,E), or total Figures 4C,F) set, patients in the high-risk group had poorer survival and higher risk scores compared with those in the low-risk group. The heatmaps manifested the differential expression pattern of PHRLs between the high- and low-risk groups (Figures 4G–I).

**FIGURE 5:**
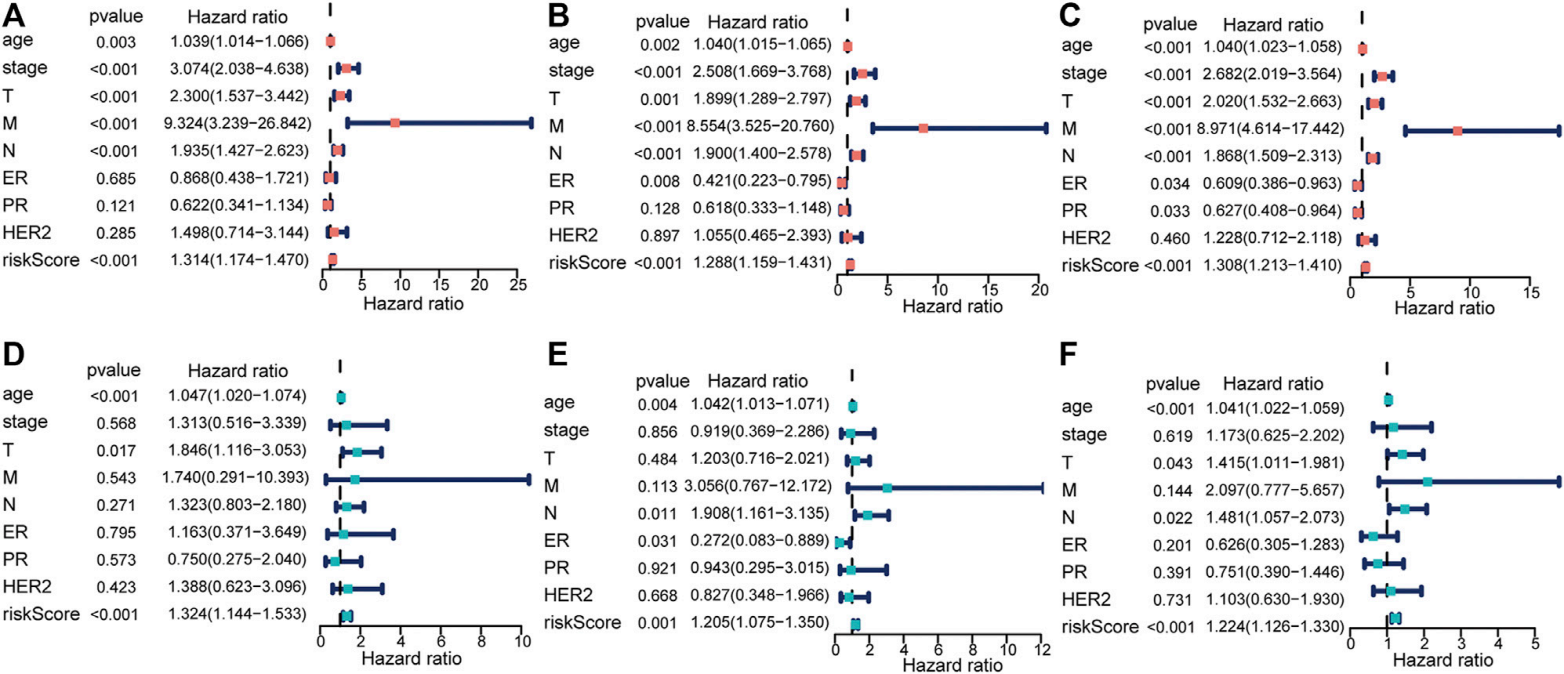
Identification of the risk score as an independent prognostic factor by Cox regression analysis. **(A,D)** Training set. **(B,E)** Testing set. **(C,F)** Total set.

We excluded 305 patients with incomplete clinical and pathological information to further test the independent predictive ability of the risk model. In order to ensure the accuracy of the following analyses, we compared the difference in clinicopathological factors of breast cancer patients between training (n = 383) and testing (n = 402) sets (Supplementary Table S5). As expected, there was no significant difference between the two sets randomly sampled. Then, we performed univariate and multivariate Cox regression analyses. The results showed that the risk score was significantly associated with OS (*p* ≤ 0.001) after adjustment for age, stage, TNM stage, ER/PR status, and HER2 status in the training (Figures 5A,D), testing (Figures 5B,E), and total sets (Figures 5C,F), suggesting that the risk score could act as an independent prognostic factor.

**FIGURE 6:**
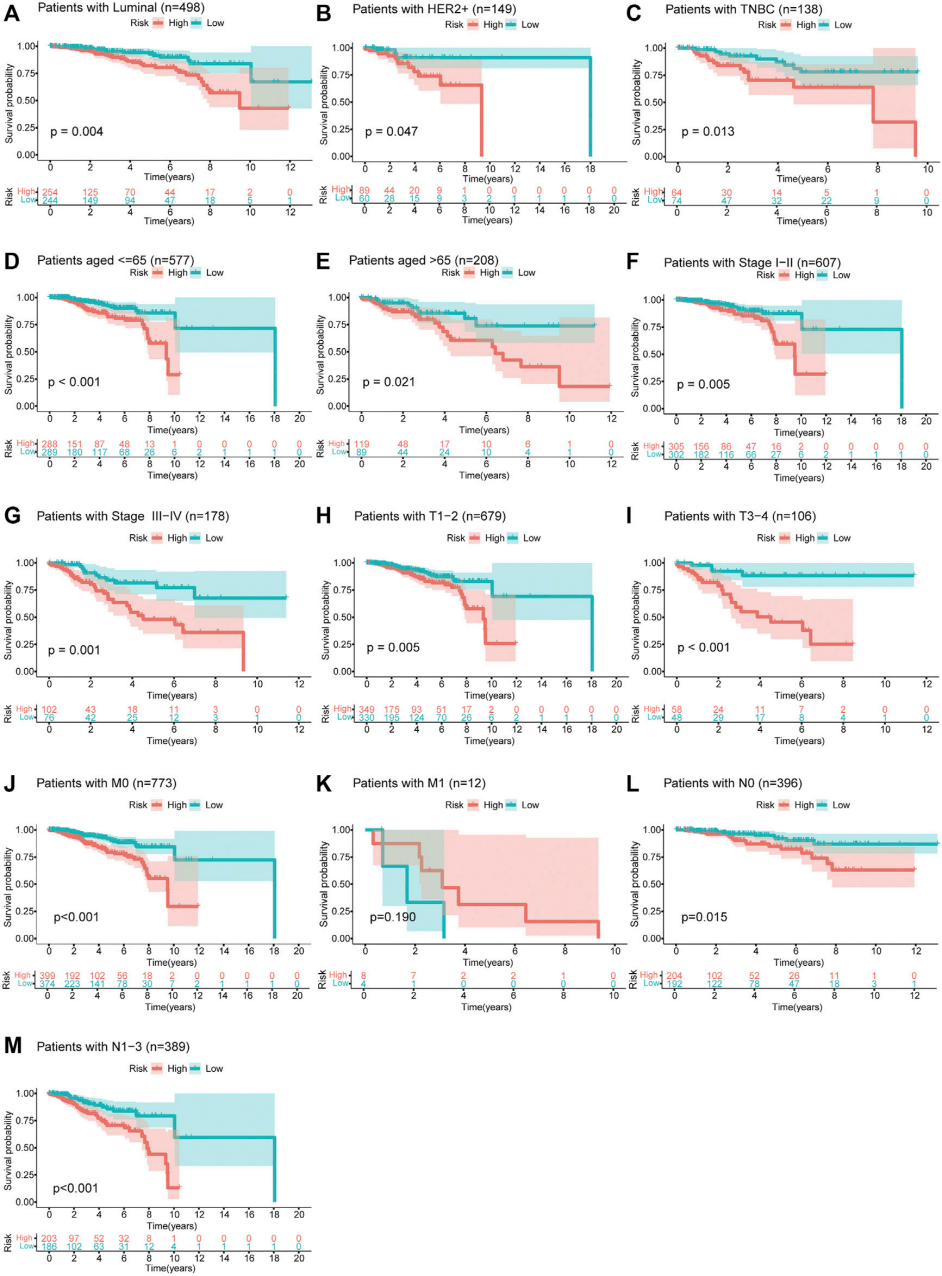
Survival curves of OS between high- and low-risk groups for patients classified in different ways. **(A–C)** Patients with HR-positive/luminal breast cancer, HER2-positive breast cancer, or TNBC. **(D,E)** Patients aged ≤65 or >65 years. **(F,G)** Patients with clinical stage I–II or III–IV. **(H,I)** Patients with T1–2 or T3–4. **(J,K)** Patients with M0 or M1. **(L,M)** Patients with N0 or N1–3.

Moreover, we divided patients in the total set into multiple groups according to their clinicopathological characteristics and then used Kaplan–Meier survival analysis to verify the prognostic value of the risk model for them. The results shown in Figures 6A–M indicated that the prognosis of patients in the high-risk group was significantly worse than that of the patients in the low-risk group. It is worth noting that this model was applicable to patients with breast cancer of three different molecular subtypes [HR-positive/luminal, HER2-positive, or triple-negative breast cancer (TNBC)]. By comparing the risk scores of patients in different groups, we found that patients over 65 years and those with higher clinical stage had higher risk scores while patients with TNBC had lower risk scores (Supplementary Figure S3).

### Establishment and Validation of a Nomogram and Drug Sensitivity Analysis

**FIGURE 7:**
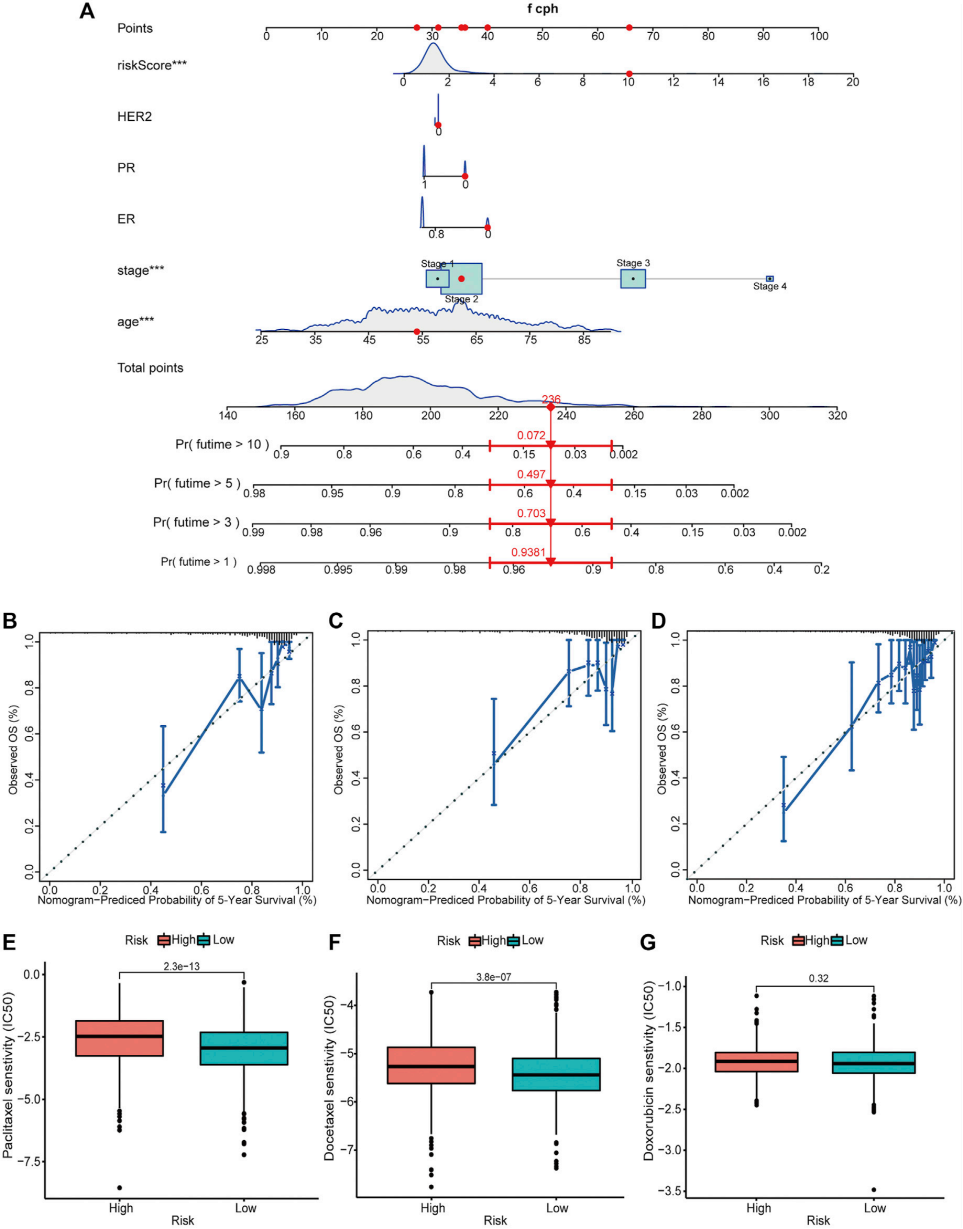
Establishment and validation of a nomogram for predicting the OS of patients with breast cancer along with the drug sensitivity analysis. **(A)** A nomogram considering multiple independent prognosticators, including age, stage, ER, PR, HER2 status, and risk score for predicting the 1-, 3-, 5-, and 10-year OS of patients in the total set. Calibration curves of the nomogram predicting 5-year OS in the training set **(B)**, testing set **(C)**, and total set **(D)**. (We did not show the nomograms of the training set and the testing set.) Drug sensitivity of patients in high- and low-risk groups to paclitaxel **(E)**, docetaxel **(F)**, and doxorubicin **(G)** in the total set. ***p < 0.001.

The independent prognosticators, including age, stage, ER, PR, HER2 status, and risk score, were used to establish a nomogram for the total set, which could assign a point for each subgroup of these prognosticators. Then, we could calculate the total points to predict the 1-, 3-, 5-, and 10-year OS. As shown in Figure 7A, a 54-year-old patient with clinical stage II, risk score of 10.02, and TNBC had a total point of 236 and predicted 1-, 3-, 5-, and 10-year OS of 93.8, 70.3, 49.7, and 7.2%, respectively. The C-index was 0.816 (95% CI 0.760–0.873) in the training set (*n* = 383), 0.800 (95% CI 0.725–0.874) in the testing set (*n* = 402), and 0.797 (95% CI 0.746–0.848) in the total set (*n* = 785). Moreover, we constructed calibration curves for three sets to predict 5-year OS, which validated the great repeatability and reliability of the established nomogram (Figures 7B–D). Intrinsic and acquired resistance to chemotherapy remains a major challenge in effective breast cancer treatment. Hence, we calculated the differences in drug sensitivity, which was decided by IC50 between high- and low-risk groups. As predicted, patients in the high-risk group had lower chemosensitivity to paclitaxel (*p* = 2.3e-13), docetaxel (*p* = 3.8e-07), and doxorubicin (not significant) compared with those in the low-risk group (Figures 7E–G).

### Functional Annotation in High- and Low-Risk Groups by GSEA

**FIGURE 8:**
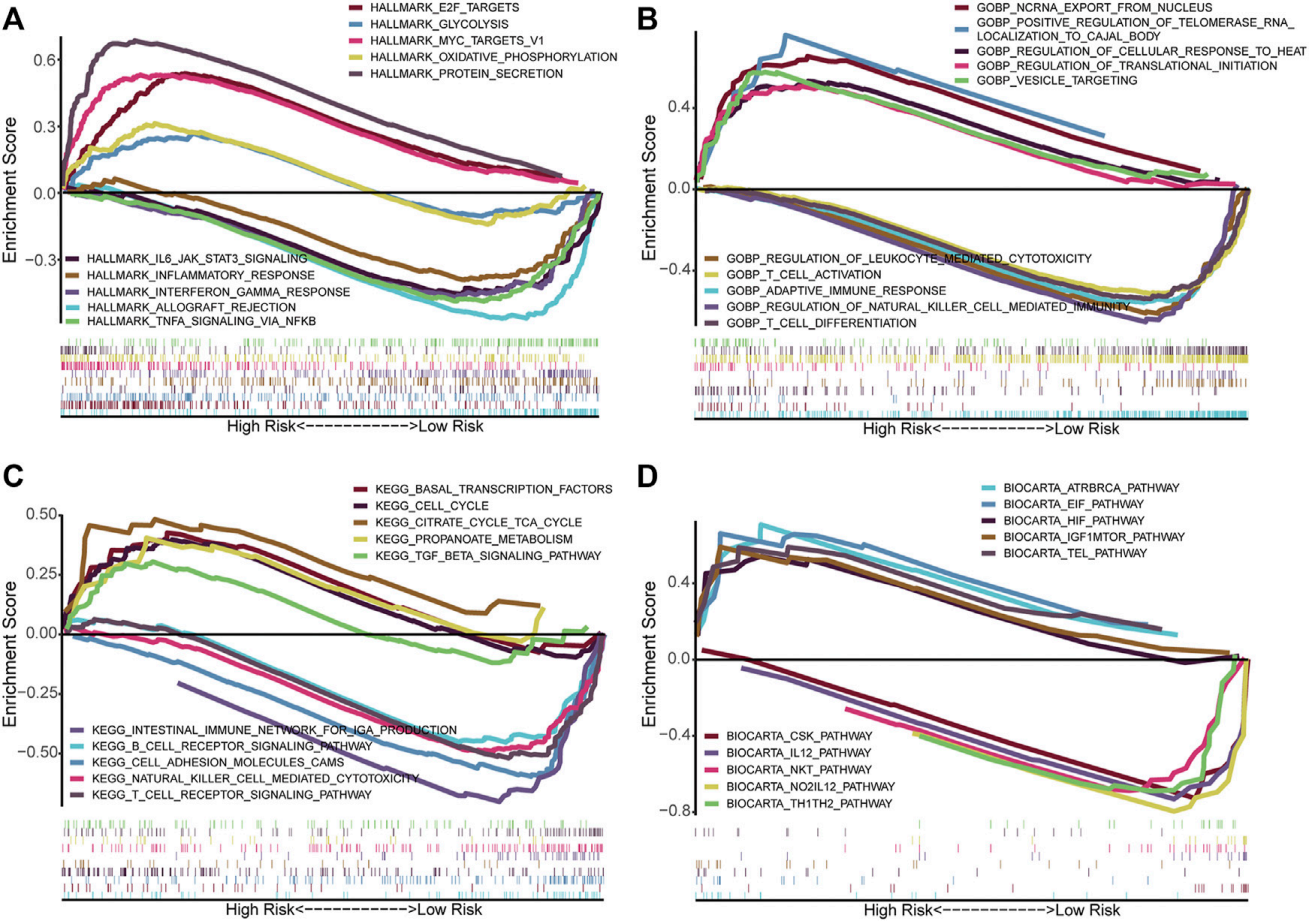
Functional annotation in high- and low-risk groups by GSEA. The GSEA results based on the gene sets, including Hallmark **(A)**, GO_BP **(B)**, KEGG **(C)**, and BioCarta **(D)**. BP, Biological process.

We performed GSEA using the gene sets, including Hallmark (Figure 8A), GO (Figure 8B), KEGG (Figure 8C), and BioCarta (Figure 8D), to identify the related differential biological function and pathways between high- and low-risk groups. Remarkably, multiple immune-related signaling pathways were enriched in the low-risk group, such as inflammatory response, interferon gamma response, TNFA signaling via NFκB, T cell activation, regulation of natural killer cell mediated immunity, T cell differentiation, T cell receptor signaling pathway, B cell receptor signaling pathway, IL12 pathway, Th1Th2 pathway, NKT pathway, and so on. Based on the GSEA results, we would like to further explore the difference in immune infiltration between the groups.

### Immune Cell Infiltration Analysis

**FIGURE 9:**
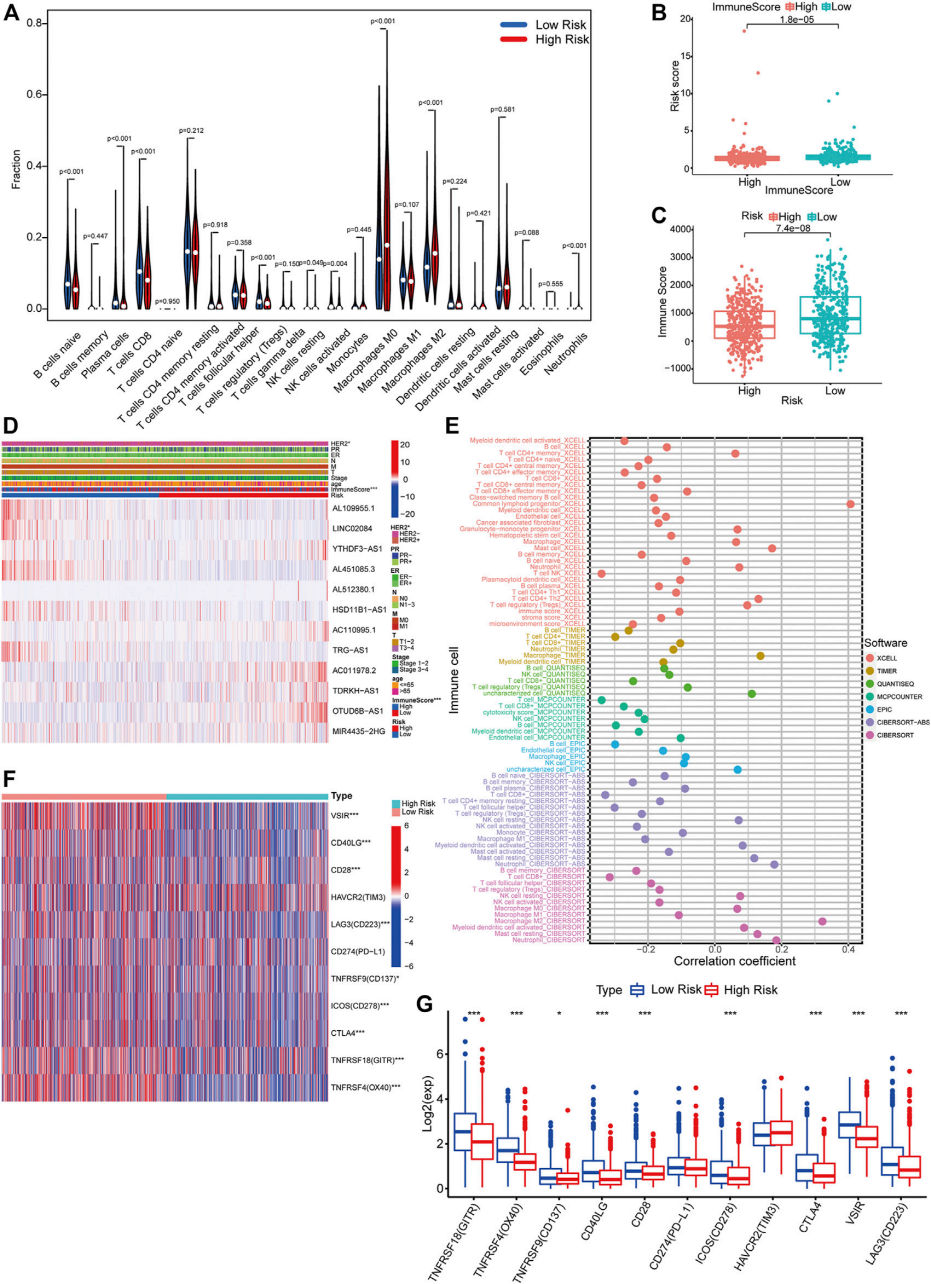
Differential immune features between risk groups. **(A)** CIBERSORT algorithm results demonstrated the differential infiltration levels of 22 kinds of immune cells for each sample in TCGA between the high- and low-risk groups (Wilcoxon test). **(B)** Comparison of immune scores of patients between the high- and low-risk groups (Wilcoxon test). **(C)** Comparison of risk scores of patients between the high- and low-immune score groups (Wilcoxon test). **(D)** A comprehensive heatmap integrating the expression of 12 PHRLs and the distribution of multiple clinicopathological factors (HER2, PR, ER, TNM, stage, age, and immune score) between the high- and low-risk groups (chi-square test). **(E)** A bubble chart exhibited the correlation between infiltrating immune cells and risk scores using CIBERSORT, CIBERSORT-ABS, TIMER, xCELL, quanTIseq, EPIC, and MCPcounter algorithms (Spearman’s rank correlation). **(F,G)** Heatmap and box plot showed the expression of 11 immune checkpoints in the high- and low-risk groups (Wilcoxon test). *p < 0.05, **p < 0.01, and ***p < 0.001.

With the CIBERSORT algorithm, we compared the differential infiltration levels of 22 kinds of immune cells for each sample between the high- and low-risk groups. As shown in Figure 9A, plasma cells (*p* < 0.001), resting NK cells (*p* = 0.049), M0 macrophages (*p* < 0.001), M2 macrophages (*p* < 0.001), and neutrophils (*p* < 0.001) were significantly enriched in the high-risk group while the proportions of naïve B cells (*p* < 0.001), CD8+T cells (*p* < 0.001), regulatory T cells (Tregs) (*p* < 0.001), and activated NK cells (*p* = 0.004) were significantly higher in the low-risk group. To validate the observed differences, we divided the patients into high- and low-immune score groups according to the median immune score. We then compared the risk scores between them (Wilcoxon test). Obviously, patients with high immune scores had lower risk scores (*p* < 0.001) (Figure 9B). Consistently, the immune scores of patients in the high-risk group were lower than those of patients in the low-risk group (*p* < 0.001) (Figures 9C,D). Additionally, we calculated the correlation between infiltrating immune cells and risk scores and exhibited it in a bubble chart (*p* < 0.05) (Figure 9E). Immune checkpoints are immunomodulators of both stimulatory and inhibitory pathways (Pardoll, 2012). We obtained 11 immune checkpoints from the previous literature (Marin-Acevedo et al., 2018) and then compared their expression level between the high- and low-risk groups. Most of them (GITR, OX40, CD137, CD40LG, CD28, CD278, CTLA4, VSIR, and CD223) were dramatically downregulated in the high-risk group (Figures 9F,G).

### Validation of the Expression of the 12 PHRLs in Breast Cancer Tissues and Breast Cell Lines

**FIGURE 10:**
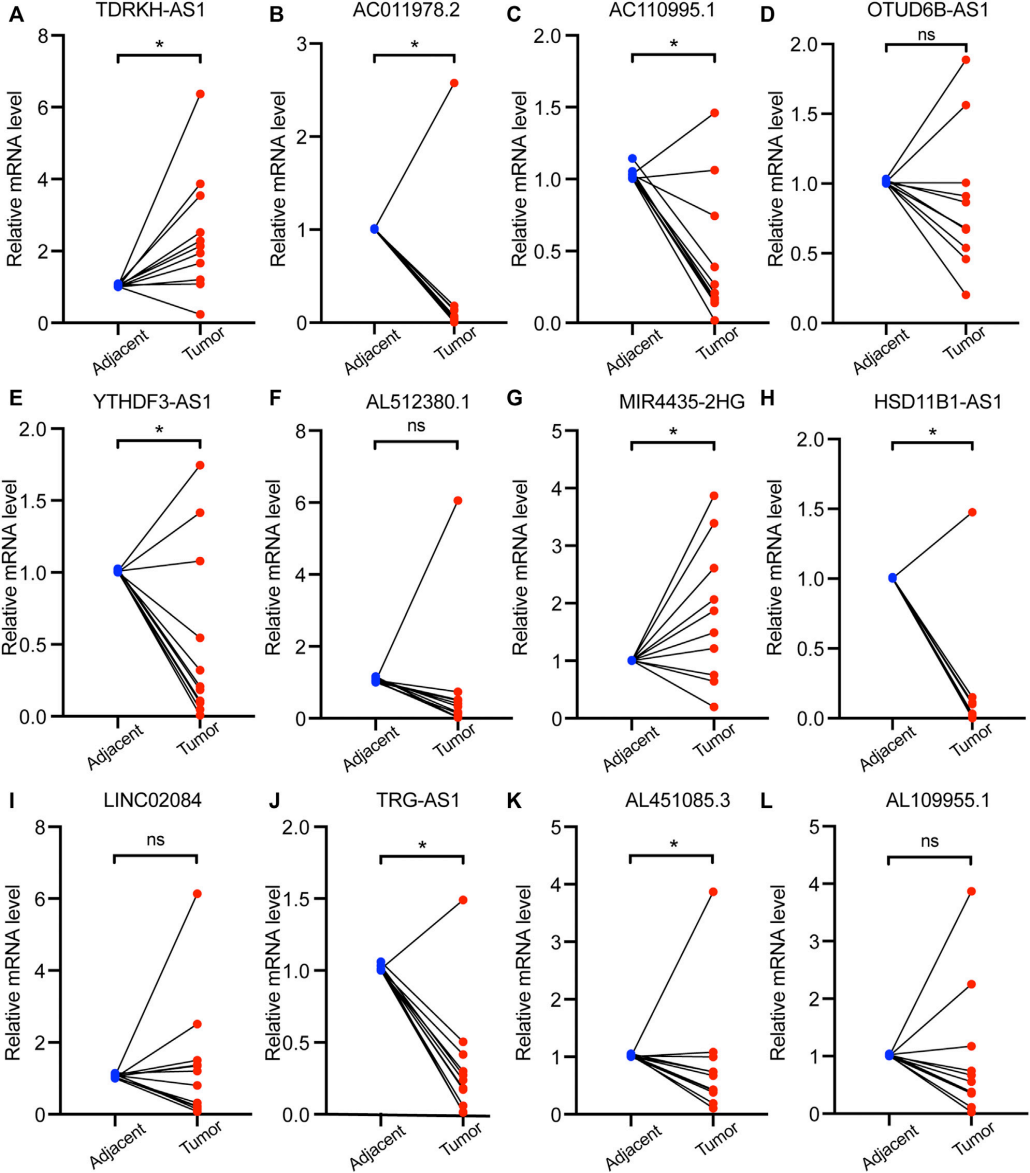
The expression of 12 PHRLs in 11 breast cancer tissues and paired normal tissues. **(A)**TDRKH-AS1, **(B)** AC011978.2, **(C)** AC110995.1, **(D)** OTUD6B-AS1, **(E)** YTHDF3-AS1, **(F)** AL512380.1, **(G)** MIR4435-2HG, **(H)** HSD11B1-AS1, **(I)** LINC02084, **(J)** TRG-AS1, **(K)** AL451085.3 and **(L)** AL109955.1.

**FIGURE 11:**
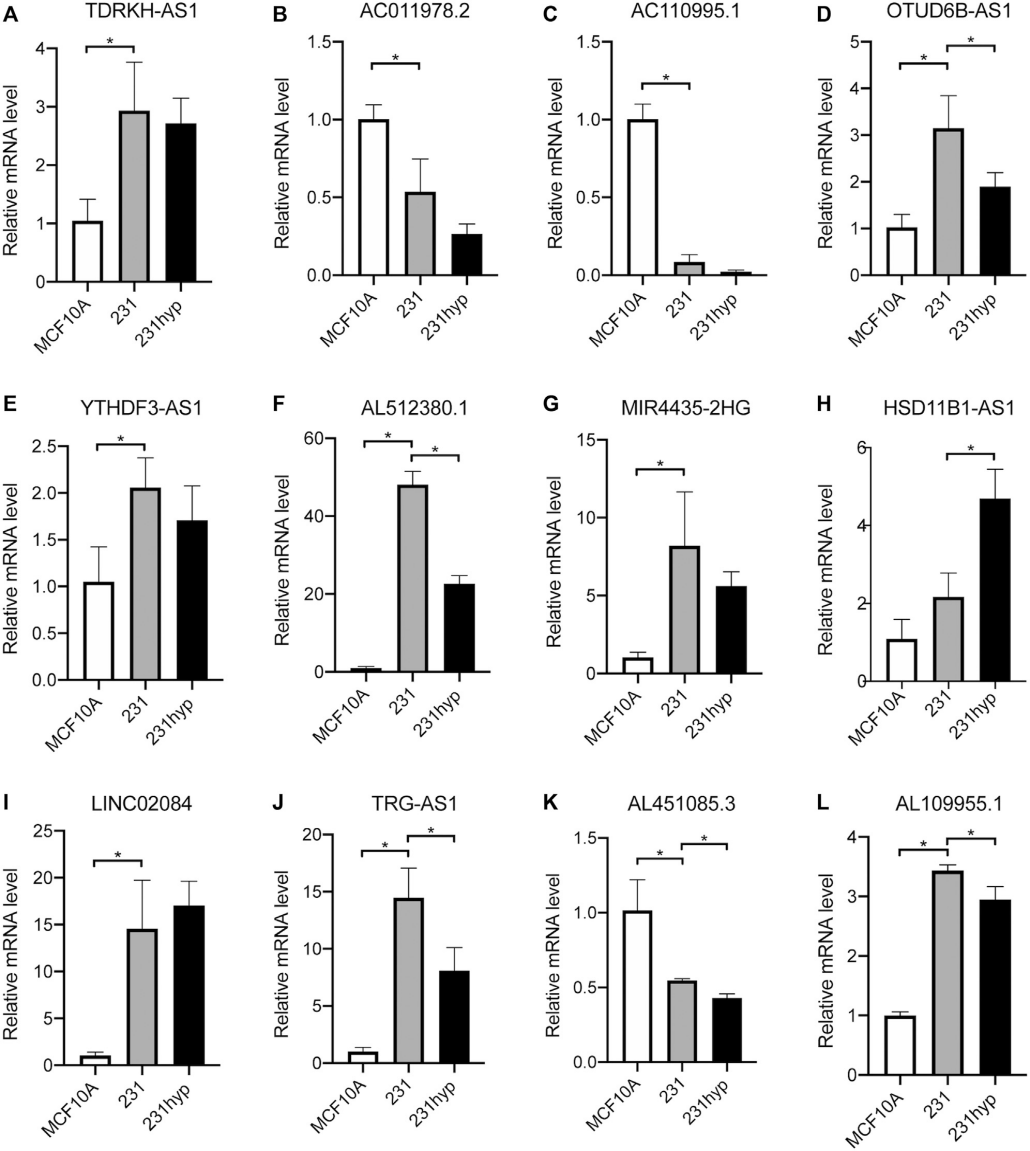
The expression of 12 PHRLs in breast cell lines. **(A)**TDRKH-AS1, **(B)** AC011978.2, **(C)** AC110995.1, **(D)** OTUD6B-AS1, **(E)** YTHDF3-AS1, **(F)** AL512380.1, **(G)** MIR4435-2HG, **(H)** HSD11B1-AS1, **(I)** LINC02084, **(J)** TRG-AS1, **(K)** AL451085.3 and **(L)** AL109955.1. hyp: hypoxia (1% O_2_) treatment for 24 h.

**FIGURE 12:**
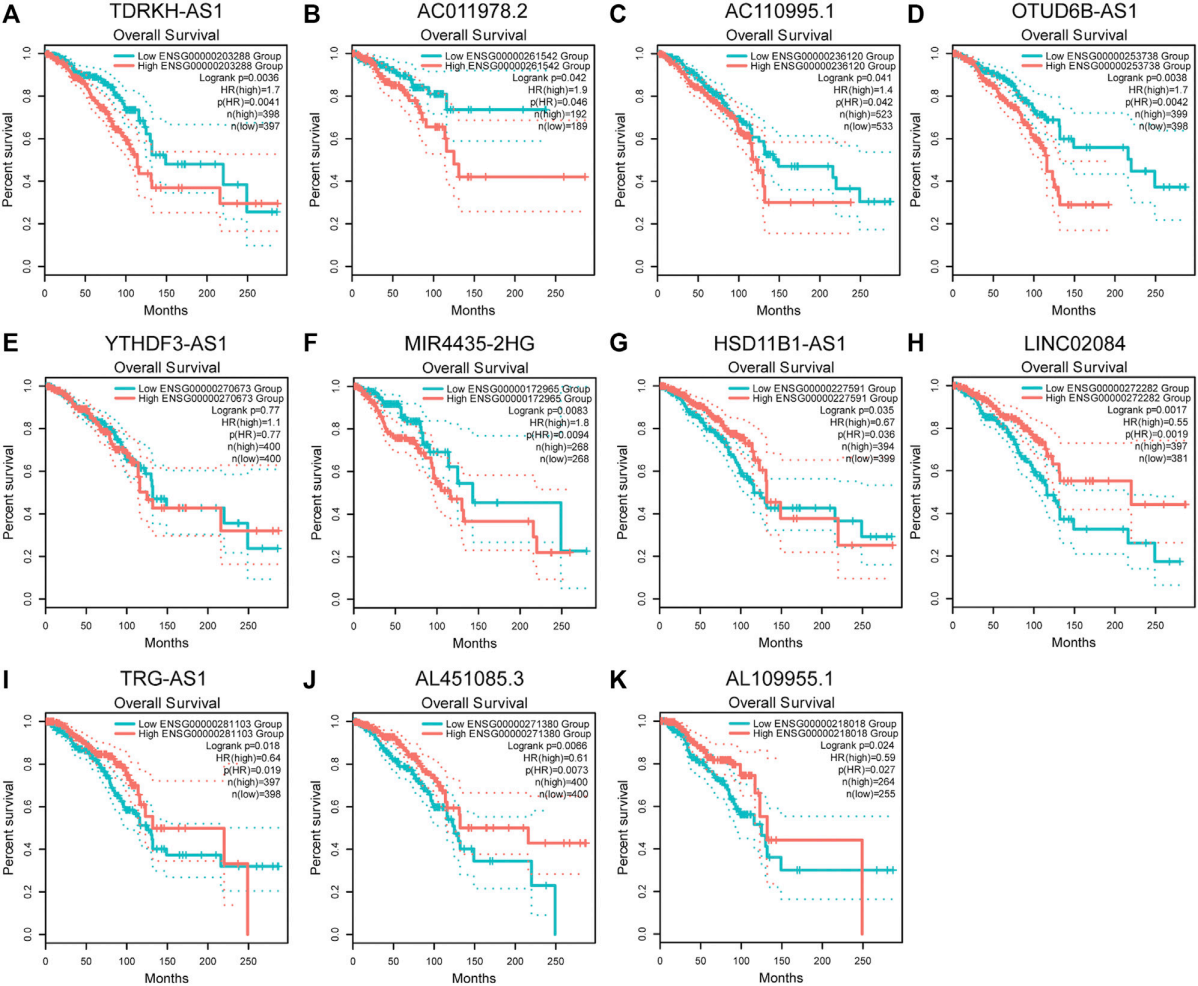
Kaplan-Meier survival analysis of breast cancer patients based on the expression of PHRLs using GEPIA2 database. **(A)** TDRKH-AS1, **(B)** AC011978.2, **(C)** AC110995.1, **(D)** OTUD6B-AS1, **(E)** YTHDF3-AS1, **(F)** MIR4435-2HG, **(G)** HSD11B1-AS1, **(H)** LINC02084, **(I)** TRG-AS1, **(J)** AL451085.3 and **(K)** AL109955.1.

To verify the differential expression of the 12 PHRLs, 11 breast cancer tissues and the corresponding adjacent normal tissues were collected for RT-qPCR assay. Two lncRNAs were significantly overexpressed in tumor tissues while six were significantly underexpressed (Figure 10). We further explored the expression of the 12 PHRLs in MCF10A (normoxia, 21%O_2_), MDA-MB-231 (normoxia, 21%O_2_), and MDA-MB-231 (hypoxia, 1%O_2_). The results were shown in Figure 11. We next carried out the Kaplan–Meier survival analysis for the 12 PHRLs using GEPIA2 database (http://gepia2.cancer-pku.cn/) except AL512380.1 for its median expression being zero (Figure 12). Based on the transcriptomic data from TCGA and the Genotype-Tissue Expression (GTEx) database, we analyzed the differential expression of the 12 PHRLs between breast cancer tissues or TNBC tissues and normal breast tissues (Supplementary Figures S4 and S5). Taken together, we could also draw a conclusion that TDRKH-AS1, MIR4435-2HG, HSD11B1-AS1, TRG-AS1, and AL451085.3 were valuable prognostic indicators for patients with breast cancer.

## Discussion

Breast cancer remains the most common cancer worldwide, with continuously increasing incidence and various factors affecting its development (Bray et al., 2015; Loibl et al., 2021b). Hypoxic areas are distributed heterogeneously throughout the tumor mass and surrounding environment. The crosstalk between tumor cells and the local microenvironment contributes to carcinogenesis, metastasis, and chemoresistance and even determines clinical outcomes. Over the past decade, an increasing number of lncRNAs have been verified to play crucial roles under hypoxia TME in breast cancer. For example, lncRNA BCRT1, which is transcriptionally regulated by HIF-1α under hypoxic conditions, promoted breast cancer cell proliferation and progression (Byrne et al., 2020). Interestingly, another study showed that hypoxia-induced lincRNA-P21 promoted the Warburg effect to increase ATP generation by regulating HIF-1α transcriptional activity in breast cancer (Yang et al., 2014). Most of these studies focused on the role of lncRNAs in the occurrence and development of breast cancer.

Although several articles concerning prognostic models in breast cancer have been published (Li et al., 2020; Pei et al., 2020; Zhao et al., 2021), in this study, we selected a well-recognized gene set “HALLMARK_HYPOXIA” which consists of 200 canonical hypoxia-related mRNAs to perform a differential expression analysis between breast cancer tissues and normal tissues and obtained 46 DEHmRNAs. The co-expression analysis was performed to screen out 166 HRLs. Using these HRLs, we finally constructed a novel prognostic model for patients with breast cancer, which consisted of 12 PHRLs: TDRKH-AS1, AC011978.2, AC110995.1, OTUD6B-AS1, YTHDF3-AS1, AL512380.1, MIR4435-2HG, HSD11B1-AS1, LINC02084, TRG-AS1, AL451085.3, and AL109955.1 (RBM38-AS1). The PHRLs above were totally different from those four of Zhao et al.’s: AL031316.1, AC004585.1, LINC01235, and ACTA2-AS1. The principle reason for this difference is the different hypoxia-related gene sets we chose, which led to only 13 overlapped DEHmRNAs and 34 overlapped HRLs between our study and theirs in the co-expression network (Supplementary Table S6). Nevertheless, the KEGG analysis for the 46 DEHmRNAs we filtered out verified the accurate and strong correlation with hypoxia. Moreover, our prognostic model could divide the patients with breast cancer into high- and low-risk groups more effectively in both training and testing sets and was found to have greater prognostic value by multiple verification methods. The AUCs for 1-, 3-, 5-, and 10-year OS in three sets were calculated to evaluate the predictive accuracy. Beside OS, the difference in DFS between high- and low-risk patients was also significant. It’s also worth noting that our prognostic model was applicable to all breast cancer patients with different clinical stages or molecular subtypes (HR-positive/luminal, HER2-positve, or TNBC) while Zhao et al.’s model applied only to early-stage breast cancer patients. When we compared the risk scores of patients in different groups, we found that patients over 65 years or those with later clinical stage had higher risk scores while patients with TNBC had lower risk scores. There may be two reasons accounting for this result: For one thing, among the 208 patients over 65 years in TCGA breast cancer database, only 21 of them were with TNBC; for another, there were merely 23 TNBC patients among a total of 178 patients with late stage (stage III or stage IV). This seems to indicate that the roles of age and clinical stage as clinicopathological factors outweigh the role of molecular subtypes to predict the survival of patients in the current study, which has also been proved by previous studies (Ferguson et al., 2013; O'Brien et al., 2018). The nomogram established in the present study, which took the clinicopathological factors (HER2, PR, ER, stage, and age) and risk scores into consideration, could precisely predict the OS of patients with breast cancer. The drug sensitivity analysis showed that patients in the high-risk group may be more likely to be resistant to chemotherapy drugs including paclitaxel, docetaxel, and doxorubicin. Though we failed to retrieve ideal GEO datasets including all the 12 PHRLs for independent validation, the validation process above was still sufficient to underscore the utility of our model in predicting the prognosis of breast cancer patients.

Among the 12 PHRLs, AC011978.2, AC110995.1, YTHDF3-AS1, AL512380.1, HSD11B1-AS1, AL451085.3, and AL109955.1 have not been reported to date. TDRKH-AS1 was reported to promote colorectal cancer (CRC) progression through the Wnt/β-catenin signaling pathway (Jiao et al., 2020). In addition, its differential expression with copy number alteration was positively associated with longer OS in lung adenocarcinoma (Wang L. et al., 2019). OTUD6B-AS1 was demonstrated to indicate poor prognosis in ovarian cancer, clear cell renal cell carcinoma (ccRCC), and breast cancer (as an immune-related lncRNA) while its overexpression inhibited ccRCC proliferation (Wang G. et al., 2019; Li and Zhan, 2019; Ma et al., 2020). Similarly, OTUD6B-AS1 played a tumor-suppressive role in thyroid carcinoma (Wang Z. et al., 2020), bladder carcinoma (Wang Y. et al., 2020), and CRC (Cai et al., 2021; Wang et al., 2021). In contrast, in hepatocellular carcinoma (HCC), OTUD6B-AS1 could enhance cell proliferation and invasion ability via the GSKIP/Wnt/β-catenin signaling pathway (Kong et al., 2020). MIR4435-2HG has been verified to be tumor supportive in multiple forms of cancer including breast cancer (Chen D. et al., 2021). Linc02084, as a low-risk immune-related lncRNA in ccRCC(Sun Z. et al., 2020), could also be used to construct a classifier for predicting early recurrence in HCC after curative resection (Lv et al., 2018). TRG-AS1 acted as a molecular sponge to stimulate tongue squamous cell carcinoma (He et al., 2020), HCC (Sun X. et al., 2020), and glioblastoma (Xie et al., 2019) progression. By means of both bioinformatic analysis and experimental validation, our research also indicated that TDRKH-AS1, MIR4435-2HG, HSD11B1-AS1, TRG-AS1, and AL451085.3 were likely to play tumor-supportive or tumor-suppressive roles in the development and progression of breast cancer and may be valuable independent prognostic indicators for patients with breast cancer.

Immune escape has emerged as a key mechanism for breast cancer progression and a crucial step in the preinvasive-to-invasive transition (Gil Del Alcazar et al., 2020). The hypoxic areas in solid tumors are highly infiltrated with immunosuppressive cells, such as tumor-associated macrophages (TAMs), myeloid-derived suppressor cells, and Tregs (Noman et al., 2014). Liang et al. showed that hypoxia-induced exosomal lncRNA BCRT1 contributed to M2 phenotype polarization of TAMs and enhanced its tumor-promoting function (Liang et al., 2020). Ben-Shoshan et al. revealed that hypoxia induced the differentiation of nonspecific CD4^+^ T cells into functionally active Foxp3 + CD4^+^CD25^+^ Treg cells to initiate an anti-inflammatory program via HIF-1α(Ben-Shoshan et al., 2008). Neutrophils with HIF2A gain of function displayed a reduction of apoptosis both *ex vivo* and *in vivo (* Thompson et al., 2014). The presence of CD8^+^ T cells in breast cancer is a reliable predictor of clinical outcome and treatment response (Ali et al., 2014; Byrne et al., 2020). By univariable analysis, Denkert et al. found that high tumor-infiltrating lymphocytes predicted longer disease-free survival in patients with HER2-positive breast cancer and TNBC treated with neoadjuvant therapy (Denkert et al., 2018). Our GSEA results showed that multiple immune-related signaling pathways were significantly enriched in the low-risk group. To validate these findings, we performed immune cell infiltration analysis. Obviously, patients with high immune scores had lower risk scores, indicating better prognosis, in line with the findings of Bruni et al. (2020), Jiang et al. (2019), Mlecnik et al. (2016), and Savas et al. (2016). Naïve B cells, CD8+T cells, activated NK cells, and Tregs were significantly enriched in the low-risk group, which was also partially consistent with the aforementioned previous findings. Considering the importance of immune checkpoint inhibitor–based immunotherapies, we further investigated the differences in the expression of 11 immune checkpoints between the high- and low-risk groups. We found that the expression GITR, OX40, CD137, CD40LG, CD28, CD278, CTLA4, VSIR, and CD233 were downregulated in the high-risk group, which corroborated the results of the study of Hu et al. that upregulated immune checkpoint genes were positively associated with high immune infiltration and favorable prognosis in patients with invasive breast carcinoma (Hu et al., 2020). The immune cell infiltration analysis based on our prognostic model suggests that patients in the low-risk group (with high prevalence of tumor-infiltrating cells lymphocytes, elevated immune-related signaling, and high mRNA expression levels of immune checkpoints) may benefit from immune checkpoint inhibitors while patients in the high-risk group (with low prevalence of tumor-infiltrating cells lymphocytes, downregulated immune-related signaling, and low mRNA expression levels of immune checkpoints) may not. Whether and how the PHRLs influence the immune microenvironment of breast cancer still remains to be explored.

## Conclusion

In conclusion, we developed a novel prognostic model consisting of 12 hypoxia-related lncRNAs and an integrative nomogram that could predict the OS accurately and effectively for patients with breast cancer. Furthermore, we analyzed the immune cell infiltration conditions and drug sensitivity between high- and low-risk breast cancer classified based on the prognostic model. Our study uncovered dozens of potential prognostic biomarkers and therapeutic targets concerning the hypoxia signaling pathway in breast cancer.

## Data Availability

The datasets presented in this study can be found in online repositories. The names of the repository/repositories and accession number(s) can be found in the article/Supplementary Material.

## Abbreviations

LncRNA, Long noncoding RNA; TCGA, The Cancer Genome Atlas; GSEA, Gene Set Enrichment Analysis; GO, Gene Ontology; KEGG, Kyoto Encyclopedia of Genes and Genomes; OS, Overall survival; DFS (disease-free survival); ER, Estrogen receptor; PR, Progesterone receptor; HR, Hormone receptor; HER2, Human epidermal growth factor receptor 2; TNBC, Triple-negative breast cancer; LASSO, Least absolute shrinkage and selection operator; ROC, Receiver operating characteristic; AUC, Area under the ROC; HRL, Hypoxia-related lncRNA; PHRLs, Prognostic hypoxia-related lncRNAs; DEHmRNAs, Differentially expressed hypoxia-related mRNAs.
