# Supplementary material for: Development and Validation of a Novel Hypoxia-Related Long Noncoding RNA Model With Regard to Prognosis and Immune Features in Breast Cancer
Source: Front Cell Dev Biol. 2021 Dec 16;9:796729. doi: 10.3389/fcell.2021.796729 (PMC8716768; doi:10.3389/fcell.2021.796729)
Supplement: Supplementary file 1 [file Table5.pdf]

Supplementary Table 4 | Clinicopathological factors of breast cancer patients in training (n=383) and testing (n=402) sets.

Variables	TRAINING SET (N=383)	TESTING SET (N=402)	*P* value
**Age**	58.4±12.6	57.3±13.0	0.233[a]
**Pathologic T Stage**			0.304[b]
T1	102	107	
T2	238	232	
T3	34	48	
T4	9	15	
**Pathologic N Stage**			0.134[b]
N0	208	188	
N1	117	134	
N2	38	49	
N3	20	31	
**Pathologic M Stage**			0.933[b]
M0	377	396	
M1	6	6	
**Clinical Stage**			0.072[b]
I	75	68	
II	236	228	
III	66	100	
IV	6	6	
**Molecular subtypes**			0.262[b]
Luminal A/B	252	246	
HER2 positive	72	77	
TNBC	59	79	

305 patients with incomplete clinicopathological information were excluded.

[a]p-value by student's t-test

[b]p-value by Chi-square test
