# Supplementary figures and images for: Development and Validation of a Novel Hypoxia-Related Long Noncoding RNA Model With Regard to Prognosis and Immune Features in Breast Cancer
Source: Front Cell Dev Biol. 2021 Dec 16;9:796729. doi: 10.3389/fcell.2021.796729 (PMC8716768; doi:10.3389/fcell.2021.796729)

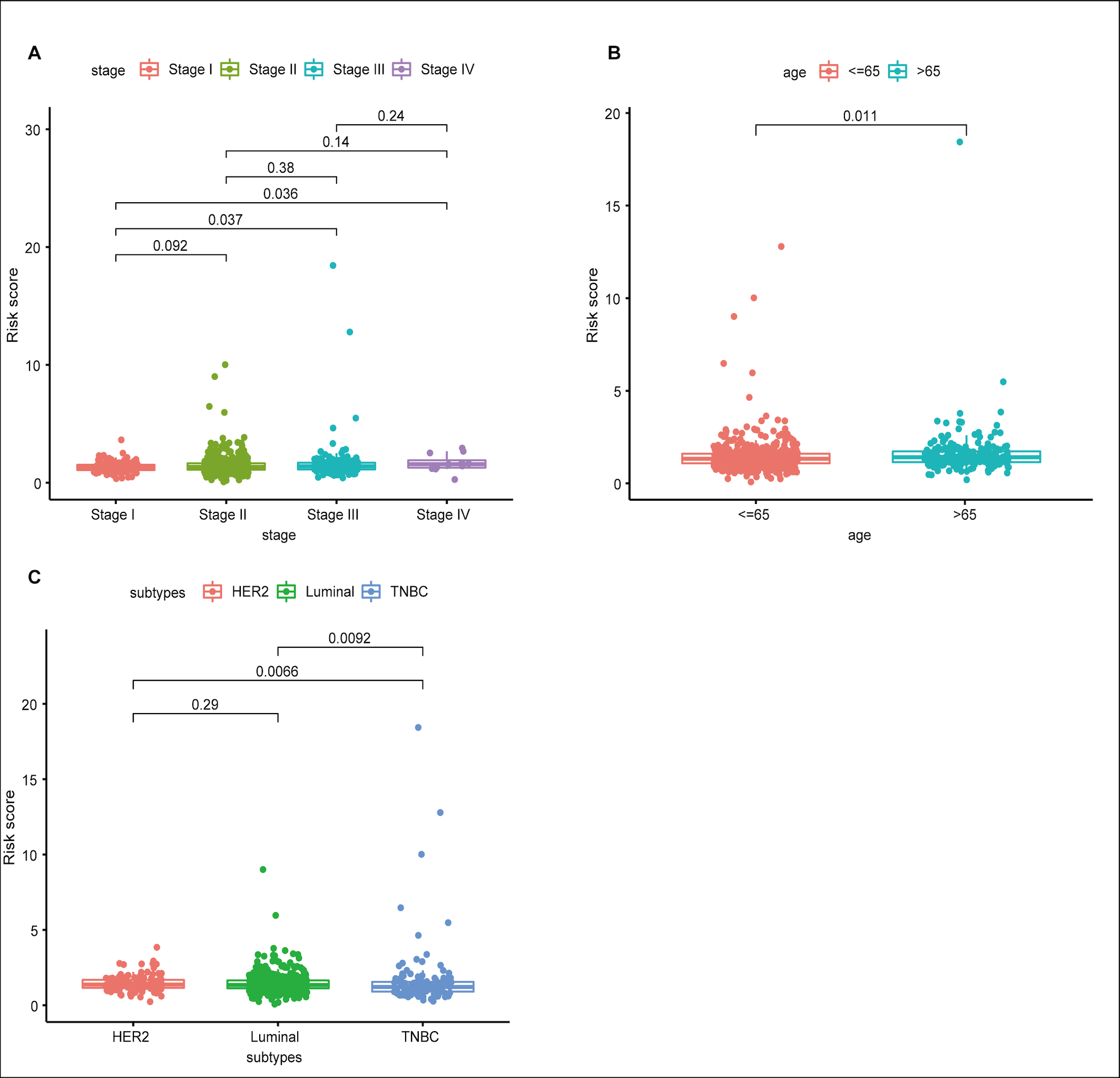

Supplement: Supplementary file 5 [file Image3.TIF]

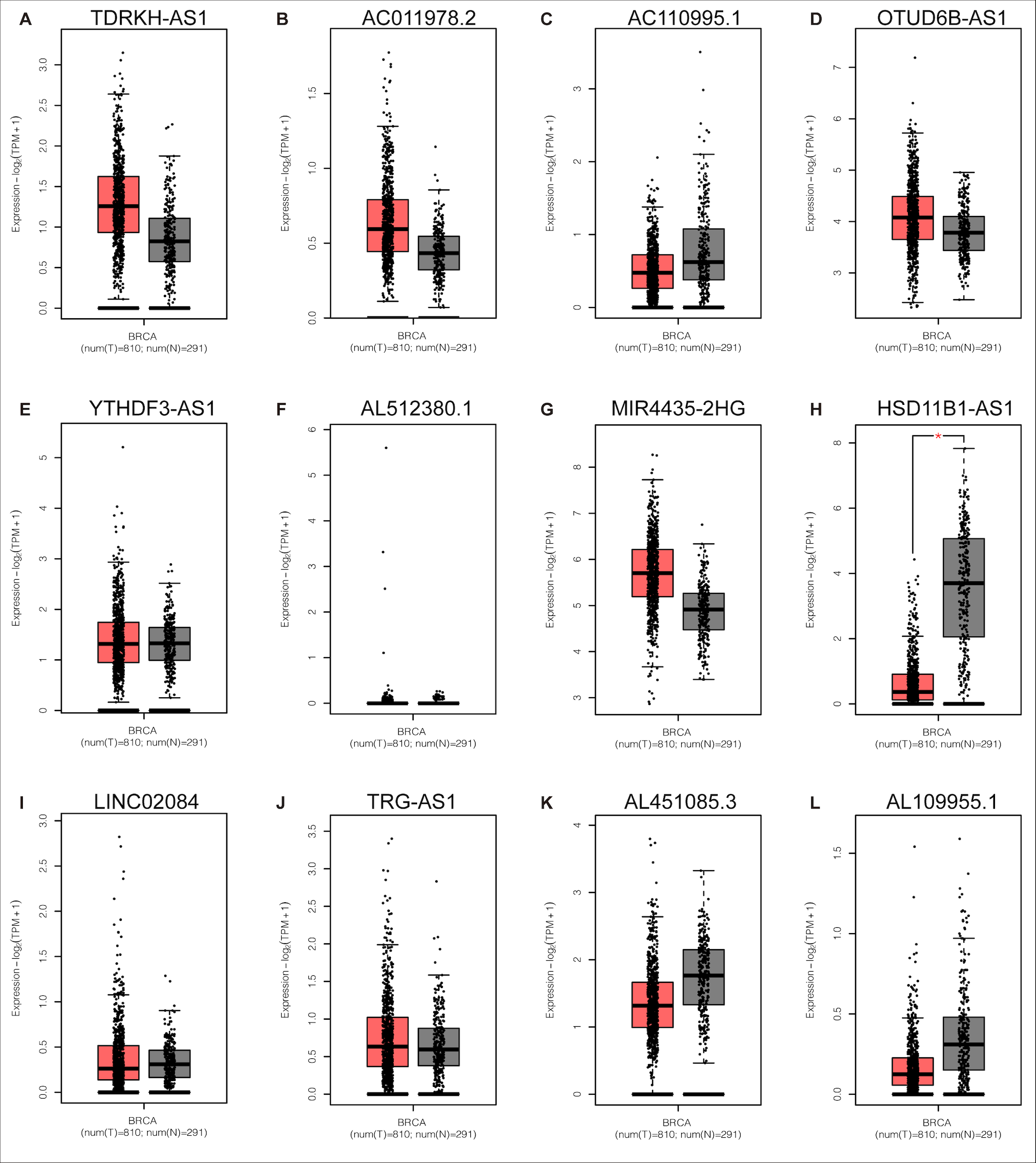

Supplement: Supplementary file 6 [file Image4.TIF]

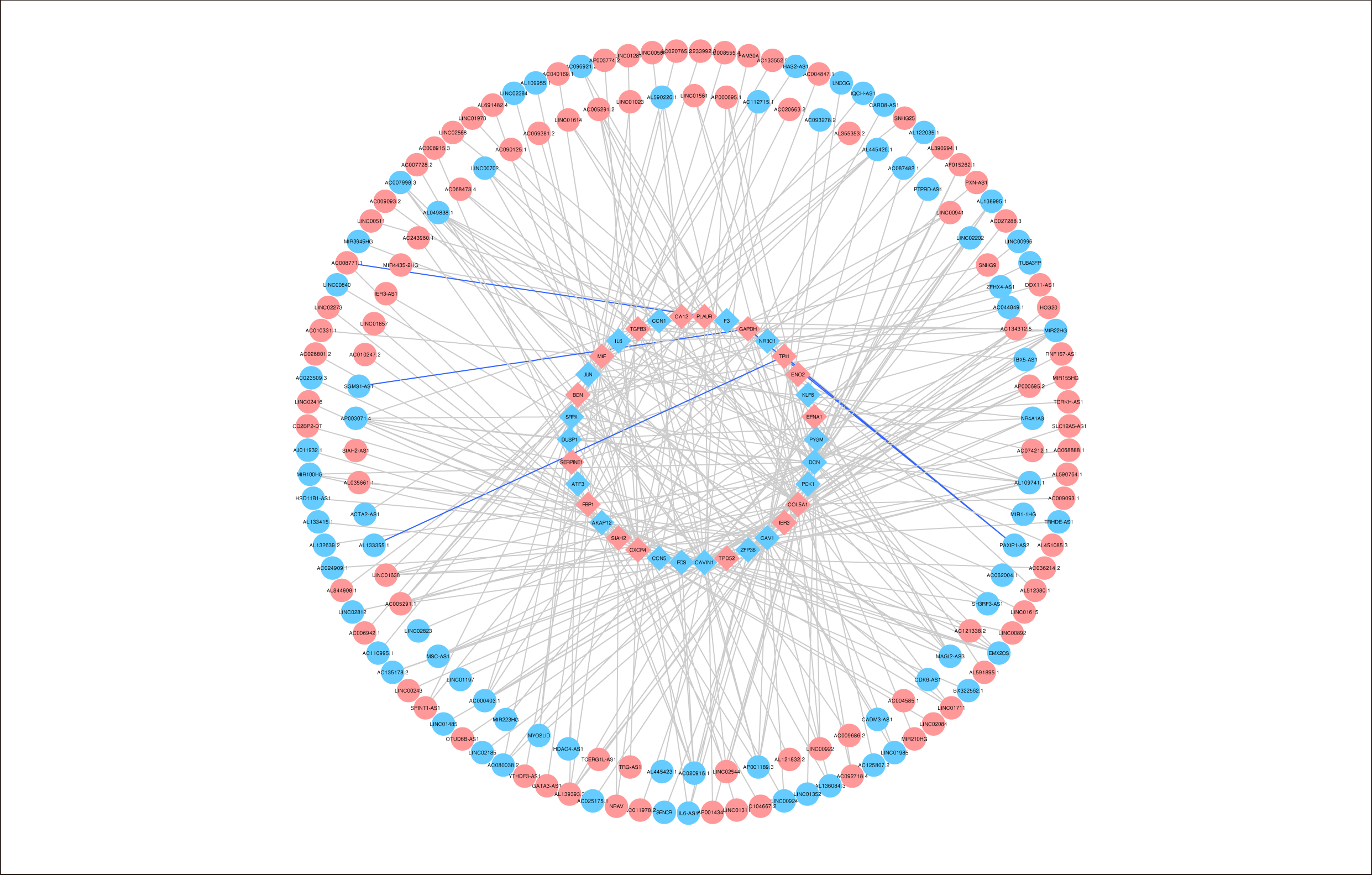

Supplement: Supplementary file 7 [file Image2.TIF]

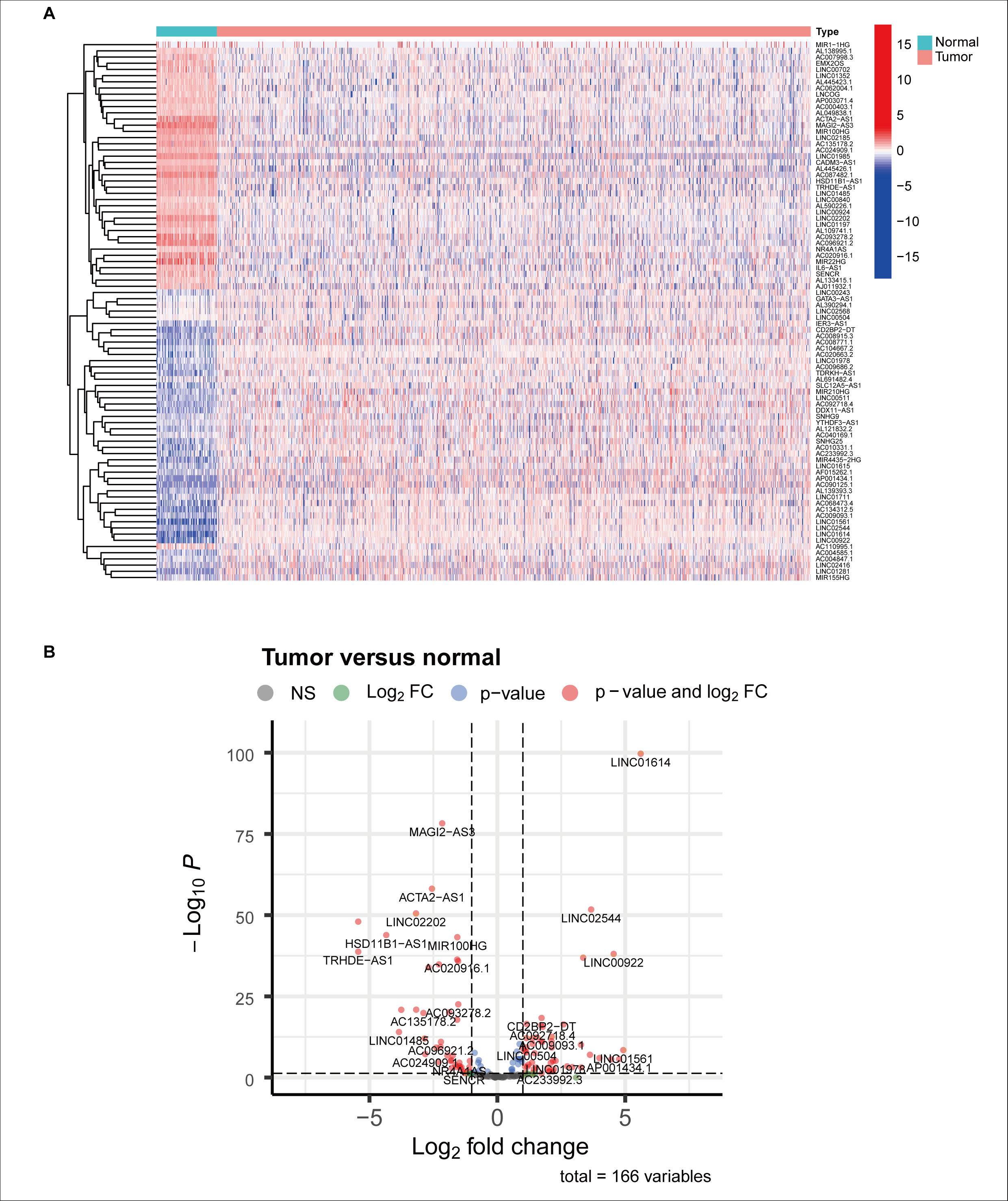

Supplement: Supplementary file 9 [file Image1.TIF]

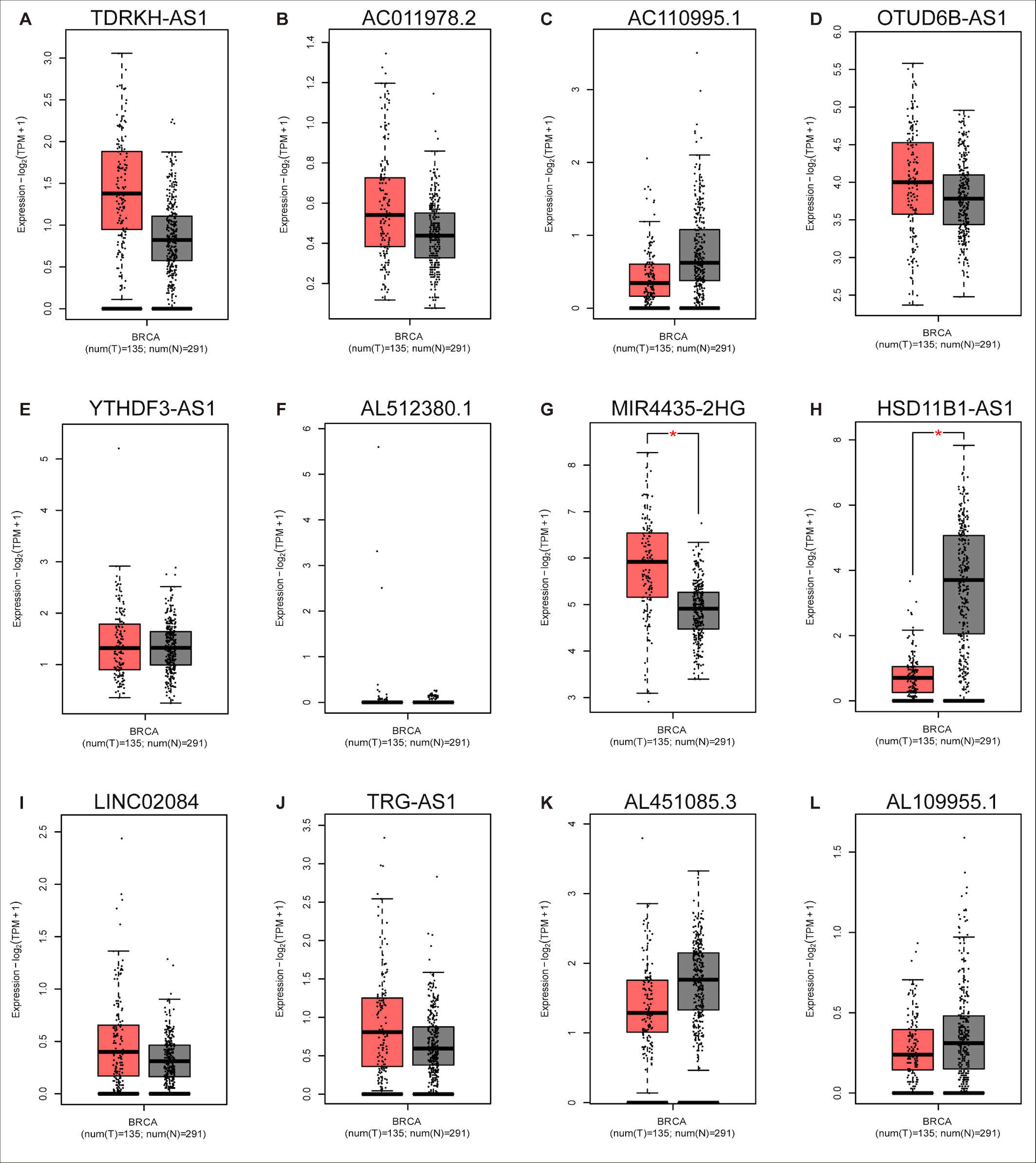

Supplement: Supplementary file 11 [file Image5.TIF]
